# Health state utilities in patients with diabetic retinopathy, diabetic macular oedema and age-related macular degeneration: a systematic review

**DOI:** 10.1186/1471-2415-13-74

**Published:** 2013-12-04

**Authors:** Edith Poku, John Brazier, Jill Carlton, Alberto Ferreira

**Affiliations:** 1 Health Economics and Decision Science, School of Health and Related Research, University of Sheffield, 30 Regent Court, Sheffield, S1 2DA, England; 2 Novartis Pharma AG, Postfach, Basel CH-4002, Switzerland

**Keywords:** Health utility, Visual acuity, Diabetic retinopathy, Age-related macular degeneration

## Abstract

**Background:**

Health state utility values (HSUVs) are important in the assessment of the cost effectiveness of new interventions. In the case of visual conditions, models generally tend have tended to be built around a set of health states defined by visual acuity (VA). The aim of this review was to assess the impact of VA on HSUVs in patients with diabetic retinopathy, diabetic macular oedema or age-related macular degeneration.

**Methods:**

A systematic literature search was undertaken in major bibliographic databases to identify articles reporting on the relationship between HSUVs and vision. Data were extracted for population characteristics, visual levels and estimated utilities. Evidence from reported statistical models, where available, was considered in the evaluation of vision in the better-seeing eye and the worse-seeing eye. Due to the heterogeneity of included studies, a narrative synthesis was undertaken.

**Results:**

Of the 17 relevant studies, 9 studies had data that could be used in the analysis of the impact of vision on HSUVs. Visual loss was associated with a marked impact on health utilities. However, the relationship was not comparable between conditions or by measure of HSUVs. Key results included the finding that overall, self-rated time-trade off estimates were more likely to discriminate between different VA levels than EQ-5D values. Additionally, a stronger correlation was observed between HSUVs and better-seeing eye VA compared to worse-seeing eye VA.

**Conclusions:**

Visual acuity has a significant impact on HSUVs. Nevertheless, care must be taken in the interpretation and use of estimates in cost-effectiveness models due to differences in measures and population diversity.

## Background

Health state utility values (HSUVs) are key parameters in the assessment of the cost effectiveness of new interventions [1]. In economic evaluation, the benefits of healthcare interventions have been commonly measured in terms of quality-adjusted life years (QALYs) where benefits are summarised into a single measure which combines length of life with health-related quality of life (HRQoL) [2]. This is achieved by assigning a value to every health state on a scale where 1 represents (usually) full health and 0 for states as bad as being dead. Negative values indicate states worse than death. There are a number of possible sources of values: patients, those at risk of developing the condition, members of the public (with or without a similar profile to the typical patient) or clinical staff.

There are three broad approaches to deriving HSUVs [3]. Firstly, preferences may be elicited using specially constructed vignettes or scenarios which describe a particular health state (such as a visual acuity category) using one of the standard health state valuation techniques such as visual analogue scaling (VAS), standard gamble (SG) or time trade-off (TTO). Secondly, a health state may be described using an existing questionnaire that is completed by patients or other proxies for which a set of values have been obtained from the general population sample. Such measures includes generic preference-based measures such as the EQ-5D, [4] SF-6D, [5] or the HUI3 [6] and condition specific preference-based measures [7] like AQL-5D for asthma [8], EORTC-8D for multiple myeloma cancer [9] and DEMQoL-U for dementia [10]. Thirdly, the preferences of the patient population towards their own current health may be measured directly, typically also using VAS, SG or TTO.

Reimbursement agencies, such as the National Institute for Health and Care Excellence (NICE) in England, now routinely assess the cost effectiveness of new health care interventions. This has resulted in a corresponding increase in the demand for data on HSUVs to populate effectiveness models. In the case of visual conditions, models are usually built around a set of health states defined by visual acuity (VA). The NICE reference case, for example, specifies that HSUVs should be derived from standardised and validated generic instruments which use a choice-based method (either TTO or SG) and should involve preferences from the general public [11]. A further recommendation specifies a preference for EQ-5D values and this has additional importance in the UK context.

Conditions such as diabetic retinopathy (DR), diabetic macular oedema (DMO) and age-related macular degeneration (AMD) have important implications for HRQoL. The changes in HSUVs as a patient moves between different VA levels are important parameters in modelling the effects of interventions that influence or modify the disease process for these conditions. Earlier reviews of utilities have focused on peripheral or central visual loss [12], the impact of eye complications in diabetic patients [13] and the psychometric properties of specific measurement tools [14].

The aim of this review was to assess the impact of VA, in the better-seeing eye (BSE) and worse-seeing eye (WSE) on HSUVs in patients with DR, DMO or AMD.

## Methods

### Literature searching

Initial searches were conducted in February 2012 in MEDLINE (Ovid MEDLINE (R) In-Process & Other Non-Indexed Citations and Ovid MEDLINE (R) 1946 to January Week 4 2012) in February 2012. Major electronic databases searched included the Cochrane Library (Issue 3); EMBASE (1980 Week 40 2009); Econlit (1969 to September 2009); CINAHL (1994 to September 2009) and Web of Science. Supplementary database searches for on-going or unpublished research were undertaken within the web-pages of the National Research Register Archive, Turning Research into Practice (TRIP) and Index to theses. Reference lists of identified reviews were also examined for relevant articles.

Both controlled and free text terms were used. Free text terms included “visual acuity”, “preference score”, “euroqol”, “time trade off” and “standard gamble”. While condition-specific terms were applied to searches in the major databases, such terms and their synonyms were combined with HSUV terms for supplementary searches. An example of the search strategy used in the MEDLINE is shown in Additional file 1.

### Study selection

Studies were included if HSUVs were elicited using direct methods (such as TTO and SG) or preference-based instruments (for example EQ-5D, HUI3 and SF-6D) in relevant populations with reported VA levels. Only full text publications in English were included; there were no other restrictions. Studies that reported utilities based on scenarios or vignettes were excluded. This review is about the association between VA and HSUVs, and while vignettes may be intended to reflect the consequences of different levels of VA, there is no measurement of VA.

### Data extraction and quality assessment

Study selection, data extraction and quality assessment was undertaken by EP and checked for accuracy and completeness by JC or JB. Disagreements were resolved through discussion. A piloted standardised form was used for data extraction. Information abstracted included study characteristics (country of study, study design, inclusion criteria), characteristics of respondents (age, gender, mean visual acuity at baseline, if provided), methods of utility elicitation, mean HSUVs and HSUVs for defined VA levels. Where available, information on regression models and coefficients of regressions were extracted.

To date, there is no consensus on recommended criteria for undertaking quality assessment of health state utility values [15]. Therefore, we assessed quality in terms of the recruitment and selection of the patients together with completion rates of the utility measures in order to assess the representativeness of the sample of the patient group and this information was used in the narrative review.

### Data synthesis and analysis

Due to expected variations in reported VA, a priori classification of VA in 4 groups based on levels reported by Brown et al. [16] was adopted to facilitate comparison of HSUVs across studies. The 'Brown’ classification [16] presented in Table 1 was adopted because it was the most widely used in the available literature. Reported VA levels were converted to Snellen units (20 feet), when required, using a conversion scale described by the International Council of Ophthalmology [17,18]. Weighted means of reported utilities of specified VA groups were then calculated. To obtain an estimate of variance between individual VA groups, the pooled variance was calculated using the formula [18]:

**Table 1:** **Visual levels according to Brown 2002 **[16]

***Description of vision***	***Visual acuity***
Good reading vision	20/20 – 20/25
Legal driving vision	20/30 – 20/40
Moderate visual loss	20/50 – 20/100
Legal blindness	≤ 20/200

This table displays the reported visual acuity classes by Brown et al. [16]. This classification was used because it was the most widely reported in the available literature and also provided meaningful interpretation of visual impairment.

$$s_{p}^{2}=\frac{\sum_{i}^{k}=1\left(\left(n_{i}-1\right)s_{i}^{2}\right)}{\sum_{i}^{k}=1\left(n_{i}-1\right)}$$

where s_p_ ^2^ = pooled variance; n_i_ = sample size of the *i*th sample; s_i_ ^2^ = variance of the *i*th sample; *k* = number of samples being combined; (*n* – 1) was used to give an unbiased estimate of the pooled variance of the mean, assuming an equal population variance.

Re-classification of VA levels was not possible for all studies. Furthermore, this was not adopted in studies reporting HSUVs by VA in the WSE. For these studies, the VA groups used in the published paper were maintained. The extent of heterogeneity in study designs and populations made it inappropriate to perform a formal meta-analysis.

## Results

**Figure 1:**
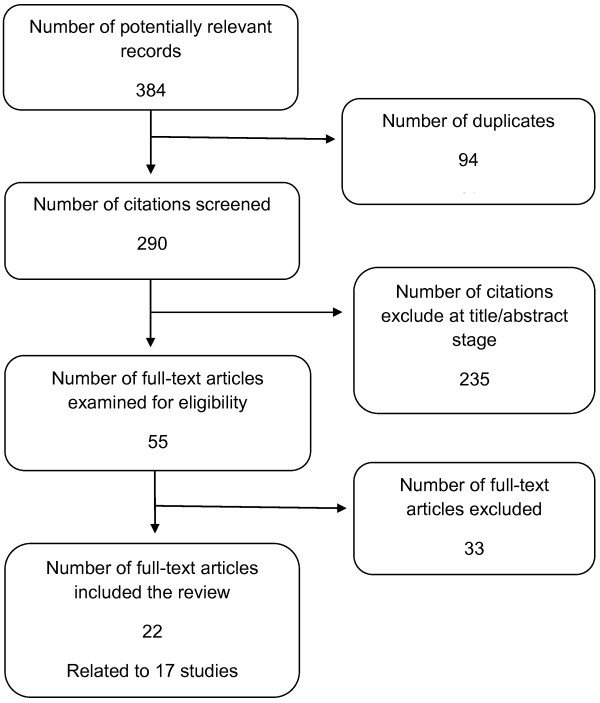
**Flow diagram of study selection.** Figure 1 is a flow diagram study selection based on pre-specified eligibility criteria. A total of 384 potentially relevant papers were identified. After de-duplication, 290 citations were screened based on their titles and available abstracts. Following the exclusion of 235 citations, 55 full text papers were retrieved for detailed examination. A total of 22 papers (relating to 17 studies) matched the eligibility criteria. Of these, one unique study involved the simulation of visual acuity levels of AMD in members of the general public. Studies that assessed ocular patients with an unspecified DR or AMD population provided information related to vision in the worse-seeing eye but were less useful for examining the relationship between HSUVs and vision. In all, 9 studies had data that could be used in the analysis of the impact of visual acuity on elicited preferences.

A flow diagram of the study selection is shown in Figure 1. Of 384 potentially relevant records, 22 full-text articles relating to 17 studies were included in this review.

### Description of included studies

No relevant study was identified for DMO patients. Overall, there were two strands of studies; studies reporting HSUVs according to VA levels in a specified population of DR or AMD patients (or a combination) [16,19-29] and studies with VA levels provided for an entire study population with ocular conditions which included patients with relevant conditions (i.e. where VA were not split by condition) [30-34]. The second group of studies provided useful information related to VA in the WSE. Of the 17 relevant studies, 9 studies [16,19,23,24,27,30,32-34] had VA data that could be re-classified into the '*Brown’* VA groups [16]. Characteristics of the study populations and methods of VA and HSUV assessments including age, gender, utility elicitation instruments, mean VA and HSUV for study participants at baseline are presented in Table 2.

**Table 2:** Summary of included studies

**Study reference, Condition, (number of patients)**	**Country**	**Mean age, years (SD)**	**Females (%)**	**Reference eye (** ***units of VA measurement*** **)**	**Mean VA at baseline (SD)**	**Health state utility measure (Anchor)**	**Mean utility at baseline**
Aspinall et al. [19]	UK	77.8	42	Binocular (distant) vision	0.49	TTO (perfect vision)	0.81
		(6.7)			(0.43)		
AMD (122)				Binocular (near) vision *(logMAR)*	0.72		
					(0.43)		
Au Eong et al. [20]	Singapore	68.1	62	BSE	NR	EQ-5D^a^	0.89
		(9.4)					
				WSE	NR	TTO (perfect vision)	0.81
AMD (338)				WVA *(logMAR)*	NR	SG (perfect vision/death)	0.86
						SG (perfect vision/binocular blindness)	0.91
Brown [30]	USA	67.5	63	BSE	NR	TTO (perfect vision)	NR
DR (107)				WSE *(Snellen units, feet)*	NR	SG (perfect health/death)	NR
AMD (107)							
Macular							
Oedema (5)							
Brown et al. [31]^a,b^	USA			WSE^c^*(Snellen units, feet)*		TTO (perfect vision)	NR
DR (28)	[Unilateral good vision group]	66	59		0.33 to 1.0		
AMD (36)		(11)					
Macular							
Oedema (2)							
	[Bilateral good vision group]	60	58		0.8 to 1.0		
		(10)					
Brown et al. [16]	USA			BSE *(Snellen units, feet)*		TTO (perfect vision)	0.74
DR (333)	DR	62.2	58		20/40		
AMD (248)		(11.8)					
	AMD	73.2	43		20/45		
		(9.8)					
Brown et al. [32]^a^	USA	67.5	61	BSE *(Snellen units, feet)*	NR	TTO (perfect vision)	0.79
		(12.2)					
DR (170)							
AMD (145)							
Czoski-Murray et al. [21]^c^	UK	32.0	51	BSE	NR	TTO (full health)	NR
		(12.5)					
AMD (108)							
Espallargues et al. [22]	UK	79.6	58	BSE	1.01	EQ-5D	0.72
		(7.5)			(0.67)		
AMD (209)				WSE	1.68	HUI-3	0.34
					(0.75)		
				Binocular (distant) vision	0.49	SF-6D	0.66
					(0.43)		
				Binocular (near) vision *(logMAR)*	0.46	TTO (full health)	0.64
					(0.88)		
Lee et al. [23]^d^	USA	75.4	50	BSE *(Snellen units, feet)*^*f*^	0.4	SG (perfect vision/death)	0.83-0.87
		(6.2)			(0.41)		
DR (58)						SG (perfect vision/unilateral blindness)	0.66-0.79
AMD (44)							
Lloyd et al. [24]	UK	NR	NR	BSE *(Snellen units, metres)*	NR	EQ-5D	NR
DR (122)							
Reeves et al. [25]	UK	NR	NR	BSE *(ETDRS letters)*	NR	SF-6D	NR
AMD (1,829)							
Sahel et al. [26]	France, Germany, Italy	77	60	BSE	0.49	HUI3	0.48
		(8.0)					
AMD (360)				WSE *(logMAR)*	1		
Shah et al. [33]^a^	USA	67.5	66	BSE *(Snellen units, feet)*	NR	TTO (perfect vision)	NR
DR/AMD(150)							
Sharma et al. [34]^a^	Canada	67.5	64	BSE *(Snellen units, metres)*	NR	TTO (perfect vision)	0.78
		(11.9)					
DR (105)						SG (perfect vision/death)	0.85
AMD (107)							
Sharma et al. [27]	Canada	63.5	48	BSE *(Snellen units, metres)*	NR	TTO (perfect vision)	NR
		(12.5)					
DR (221)							
Soubrane et al. [28]^e^	Canada, France, Germany, Spain, UK	78.1	65	BSE *(Snellen units, feet)*^f^	0.6	EQ-5D	0.65
		(6.9)			(0.7)		
AMD (401)							
Yanagi et al. [29]	Japan	75.9	85	BSE *(logMAR)*	NR	TTO (perfect vision)	0.6
AMD (48)						SG (perfect vision/death)	0.7

*Abbreviations*: *BGV* bilateral good vision, *BSE* better-seeing eye, *EQ-5D* EuroQol, *HUI3* Health Utilities Index Mark 3, *logMAR* logarithm of the minimum angle of resolution, *NR* not reported, *SF-6D* Short Form 6D, *TTO* time trade-off, *SG* standard gamble, *UGV* unilateral good vision, *WSE* worse-seeing eye, *WVA* weighted visual acuity.

^a^The study participants had different ocular conditions including DR, macular oedema (undefined) and AMD. Sample sizes presented here are based on the proportion of patients with DR, AMD or macular oedema within the study population.

^b^This study compared utilities between patients with bilateral (n = 69) and unilateral good vision (n = 81).

^c^In this study, AMD health states were simulated by using contact lenses in a healthy sample of a general population.

^d^Over 50% of patients had mild DR, while only 2 out of 10 AMD patients had moderate disease.

^e^This study compared utilities in patients with AMD and non-AMD 'controls’.

^f^Snellen units were converted to logMAR units.

In the absence of a consensus relating to quality assessment of health utility studies, representativeness of study population was considered as a key feature [14]. A descriptive summary of quality assessment is presented in Additional file 2. Patient recruitment resulted in a relevant selection of patients in all but one study [21] that included a sample from the general UK population. While most studies provided sufficient information on the type of visual condition (e.g. AMD or DR) considered, there was a general lack of detail to confirm the ophthalmic diagnosis or the presence of associated visual co-morbidities. The proportion of the study population that completed utility elicitation was indicative of the response rate in each study. Whereas a response rate of 46% (n = 209/451) was reported in one study of AMD patients, [22] on average, response rate across remaining studies was 97%. However, these response rates are hard to interpret due to insufficient or missing information on withdrawals, drop-outs or non-responders [19,26,28]. Overall, study quality based on sample representativeness and response rate, was considered to be satisfactory for studies that contributed data to examine the impact of VA on HSUVs [16,19,23,24,27,30,32-34] (see Additional file 2).

### Visual acuity and health utility state values

Reporting of VA levels varied across studies. Visual impairment was based on VA in better-seeing eye (BSE) [16,20-24,27-34], worse-seeing eye (WSE) [20,22,26,30,31], binocular distance visual acuity [19,22] and weighted visual acuity (WVA) [20] defined as weighted average of VA in both eyes. HSUVs reported in included studies were EQ-5D, [20,22,24,28] SF-6D, [22,25] HUI-3, [22,24,26] TTO [16,20-22,27,29-34] and SG [20,23,29,30].

Overall, generic EQ-5D estimates were found to be largely unresponsive to differences in VA levels [22]. By contrast, TTO estimates generally displayed a more consistent reduction across VA levels. However, SG-based utility estimates tended to be higher than TTO estimates in the same patients and these in turn were higher than the values from generic instruments. Furthermore, shifts in utility estimates across VA levels did not always exhibit a definitive pattern across consecutive levels of visual impairment.

Reported HSUVs according to VA in the BSE are shown in Table 3. In DR patients, TTO estimates decreased with worsening VA in the BSE; [16,19,23,24,27,30,32-34] with the greatest reduction occurring between moderate visual loss (20/50 to 20/100) and legal blindness (≤ 20/200). EQ-5D and HUI3 (mean ± SD) estimates for good reading vision to VA corresponding to counting fingers or worse ranged from ranged from 0.75 ± 0.23 to 0.34 ± 0.36 and 0.78 ± 0.22 to 0.37 ± 0.00, respectively [24]. The observed greater disutility in patients with worsening vision was explained by an anticipated or greater dependence on others [24]. Alternatively, this pattern may also have resulted from the small sample sizes of patients (n < 20 for all groups below good reading vision).

**Table 3:** Utility estimates according to visual acuity in the better-seeing eye

**Study reference**	**HSUV instrument**	**Mean utility (SD)**	**Visual acuity**			
			***20/20 to 20/25***	***20/30 to 20/40***	***20/50 to 20/100***	**≤ 20/200**
			**Mean (SD)**	**Mean (SD)**	**Mean (SD)**	**Mean (SD)**
			**N**	**N**	**N**	**N**
**Studies with visual acuity levels based on relevant conditions**						
***DR studies***						
Lloyd et al. [24]	EQ-5D	NR	0.75(0.23)	0.50(0.30)	0.68(0.29)	0.53(0.47)
			68	13	10	7
						0.34(0.36)^a^
						3
Lloyd et al. [24]	HUI3	NR	0.78(0.22)	0.30(0.38)	0.61(0.35)	0.52(0.50)
			68	13	10	7
						0.37(0.00)^a^
						3
Brown et al. [16]	TTO	0.79	0.86(0.17)	0.80(0.19)	0.77(0.18)	0.61(0.19)
		(0.2)	72	130	95	36
Sharma et al. [27]		0.79	0.88(0.19)	0.79(0.22)	0.73(0.26)	0.73(0.22)
		(0.2)	NR	NR	NR	NR
						0.48(0.47)^a^
						NR
***AMD studies***						
Aspinall et al. [19]^c^	TTO	0.81	0.93	0.86	0.74	0.68
						0.76^b^
Brown et al. [16]		0.74(0.23)	0.84(0.21)	0.80(0.19)	0.71(0.22)	0.59(0.22)
			60	65	57	65
Lee et al. [23]	SG^d^		0.89(0.23)	0.76(0.30)	NR	NR
			23	21		
Lee et al. [23]	SG^e^		0.86(0.24)	0.39(0.37)	NR	NR
			23	21		
**Studies with visual acuity levels not based on relevant conditions**^**f**^						
Brown [30]	TTO		0.89(0.17)	0.82(0.21)	0.74(0.21)	0.62(0.20)
			82	98	89	38
						0.46 (0.29)^a^
						18
Brown et al. [32]			0.88(0.15)	0.81(0.21)	0.72(0.21)	0.61(0.19)
			127	218	83	72
Shah et al. [33]			0.88(0.19)	0.90(0.14)	0.76(0.23)	
			71	43	22	
Sharma et al. [34]			0.91(0.15)	0.80(0.21)	0.71(0.21)	0.62(0.20)
			75	136	58	37
						0.473(0.32)
						17^a^
Brown [30]	SG		0.94 (0.12)	0.90(0.19)	0.81(0.21)	0.74(0.21)
			82	98	89	38
						0.60(0.30)
						18^a^
Sharma et al. [34]			0.95(0.11)	0.90(0.17)	0.77(0.28)	0.74(0.22)
			75	136	58	37
						0.60(0.32)
						17^a^

*Abbreviations: AMD* age-related macular degeneration, *DR* diabetic retinopathy, *EQ-5D* EuroQol values, *HUI3* Health utilities index Mark 3 values, *NR* not reported, *SD* standard deviation, *SG* standard gamble values, *TTO* time-trade-off estimates.

^a^Values for levels corresponding to counting fingers to no light perception.

^b^Values for levels corresponding to counting fingers to light perception.

^c^VA level based on binocular distance vision and not VA in the better seeing-eye. Authors stated that the difference between the two measures was not significant. SD not calculated because of missing data. Figures shown were derived from the nearest equivalent Snellen categories of VA in the relevant article.

^d^Standard gamble values based on the policy scale; anchors used were perfect vision/death.

^e^Standard gamble values based on the vision scale; anchors used were perfect vision/unilateral blindness.

^f^Relevant populations refers to patients with diabetic retinopathy and age-related macular degeneration. Macular oedema was not described in full, so it is unclear if patients had diabetic macular oedema.

Patients with AMD showed a monotonic reduction in TTO estimates with decreasing VA [22] and [21], although these VA levels could not be re-classified using the 'Brown’ groups. TTO estimates in patients with good reading vision in the study reported by Espallargues et al. [22] were lower compared to those reported by Brown et al. [16] and Aspinall et al. [19]. However, further comparisons of Brown et al., [16] and Aspinall et al., [19] with Espallargues (2005) [22] and Czoski-Murray et al. [21] are limited by differences in VA stratifications and elicitation methods (see Additional file 3). Nonetheless, there were comparable reductions in mean TTO utilities in the BSE in all 3 studies [16,19,22]. The lowest TTO estimate for good reading and the largest decline across the VA groups was observed in a population with simulated AMD states and this was probably because the values were provided by the general population who might not be able to adapt in the study [21].

Some studies used perfect vision as the upper anchor, but for policy purposes it is important to use perfect or full health as the upper anchor. The choice of the upper anchor is important since it has been shown to impact on estimated utilities. SG estimates based on the policy scale (perfect health/death) and the vision scale (perfect vision/unilateral blindness) [23] showed comparable utilities for mild to moderate visual loss (0.86 ± 0.24 to 0.89 ± 0.23) but contrastingly different estimates for patients with profound visual disability (0.39 ± 0.37 to 0.76 ± 0.30). This suggests that patients with severe visual impairment may be more willing to accept the risk of unilateral blindness compared to dying. As such, it is important to note the effect of different anchors in HSUVs elicitation, and how this may influence results of relevant cost-utility analyses.

### Better-seeing eye and worse-seeing eye

Available data [20,22,26,30,31] on the impact of vision on HSUVs in the BSE and WSE indicated that for VA levels of 20/200-20/400 to no perception of light in the WSE, TTO estimates ranged from 0.94 ± 0.13 to 0.81 ± 0.16 [31]. Brown et al., [35] reported elsewhere that correlation coefficients (Spearman rank co-efficient) for TTO values and SG values for VA in the BSE and WSE were 0.46 (p < 0.001) and 0.37 (p < 0.001), whereas coefficients of 0.27 (p < 0.001) and 0.25 (p < 0.001) were reported in relation to VA in the WSE. Sahel et al. [26] reported a likely relationship between vision in BSE (p < 0.01) or vision in WSE (p = 0.7) and HUI-3 scores. Additionally, a study of AMD patients in Singapore [20] reported a weak correlation between EQ-5D estimates and weighted visual acuity (WVA) (defined as weighted average of VA in both eyes), VA in the BSE and VA in the WSE (Pearson’s correlation coefficient, -0.305, -0.91 and -0.27 respectively). TTO values were also weakly associated with WVA (Pearson’s coefficient, -0.228) and VA in the WSE (Pearson’s coefficient, -0.237). Conversely for SG estimates based on a 'policy’ scale (perfect vision/death) and a 'modified’ scale (perfect vision/binocular blindness), the authors reported no association with WVA or VA in the BSE and WSE.

Reported regression analyses conducted with VA as a dependent variable demonstrated a significant relationship between VA in the BSE and reported TTO values (P < 0.0001 to 0.01) [30-32,34] which was absent for vision in the WSE (p =0.43) [32]. Sharma et al. [34] also identified VA in the affected eye (p < 0.01) and VA in the normal eye (p < 0.01) as significant predictors of utility. Using a mixed regression model, Reeves et al. [25] predicted changes of SF-6D values by 0.0058 and 0.116 for 5-letter and 100-letter decreases in BVCA. BCVA in the BSE, however, remained a strong predictor of SF-6D measures (p < 0.0001). Table 4 shows a summary of results of various regression analyses reported in the literature.

**Table 4:** Summary of reported multiple regression analyses in included studies

**Study**	**Type of regression model**	**Results of analysis**			**Notes**
		**Dependent variable**	**β-coefficient**	**p-value**	
			**(SE)**		
**predictors of tto values**					
Brown et al. [30]	OLS regression	VA (Snellen) in BSE	0.37	<0.0001	The following equation was developed from the model:
					*Utility value = 0.37 (VA) + 0.514*,
Brown et al. [31]	OLS regression	VA (Snellen), 1 'good’ eye	-0.0902	0.001	Significant differences in reported utility values were noted when patients with two 'good’ eyes (bilateral good vision) were compared with those with one 'good’ eye (unilateral good vision).
Brown et al. [32]	OLS stepwise model	VA (Snellen), BSE	NR	<0.0001	A significant relationship was demonstrated between decreasing vision in the BSE and decrements in utility values. This relationship was absent for VA in the WSE.
		VA (Snellen), WSE	NR	0.43	
Espallargues et al. [22]	OLS Stepwise model	Distant VA (logMAR), BSE	-0.04	0.686	An association was observed between distant VA in the BSE and TTO scores. Selection criteria for significant predictors were p < 0.1. Age and time since diagnosis were important for TTO values.
			(0.05)		
Sharma et al. [34]	OLS model	VA (logMAR), BSE	0.176	<0. 01	VA levels in both the affected eye (p < 0.01) and unaffected eye (p < 0.01) were independently associated with reported utilities. Better vision was associated with higher scores.
**predictors of sg values**					
Lloyd et al. [24]	Mixed model analysis	VA (Snellen), BSE	NR	NR	The VA levels were based on the levels of vision used in the health state cards developed for the study. The authors reported that described states were significant in predicting utility. Further analysis showed that SG values were not associated with a patient’s visual acuity level.
Sharma et al. [34]	Bivariate analysis	VA (logMAR), BSE	0.193	<0. 01	VA levels in both the affected eye (p < 0.01) and unaffected eye (p < 0.01) were independently associated with reported utilities. Better vision was associated with higher scores.
	Multivariate analyses				
**predictors of hui-3 values (global)**					
Espallargues et al. [22]	Multiple linear regression	Distant VA (logMAR), BSE	-0.12	0.226	A selection criterion of p < 0.1 was adopted for a backward stepwise regression model of relevant variables. Significant variables were contrast sensitivity, illness (es) of long duration and age.
			(0.43)		
	Univariate regression	VA (logMAR), BSE	-0.14	<0.01	
			(0.03)		
Sahel et al. [26]	Multiple regression	BSE: WSE	NR	NR	The adjusted R-squared showed that 21% of the variance in the global score was due to the VA levels [p < 0.01 (BSE); p = 0.31(WSE)].
		≥20/40: ≥ 20/200	0.6	NR	
		≥20/40: < 20/200	0.57	NR	
		<20/40: ≥ 20/200	0.41	NR	
		< 20/40: <20/200	0.42	NR	
**predictors of hui-3 values (vision dimension)**					
Espallargues et al. [22]	Univariate regression	VA (logMAR), BSE	-0.25	<0.01	
			(0.26)		
	Multivariate analyses	VA (logMAR), BSE	-0.21	<0.01	
			(0.04)		
Sahel et al. [26]	Multiple regression	BSE: WSE			Authors reported that 36% of the variance in the visual dimension of the HUI-3 score was expressed by the adjusted R-squared value [p < 0.01 (BSE); p = 0.7(WSE)].
		≥20/40: ≥ 20/200	0.75	NR	
		≥20/40: < 20/200	0.74	NR	
		<20/40: ≥ 20/200	0.42	NR	
		< 20/40: <20/200	0.37	NR	

*Abbreviations:BSE* better-seeing eye, *EQ-5D* euroQol values, *HUI-3* health utilities index mark3, *logMAR* logarithm of minimum angle of resolution, *NR* not reported, *OLS* ordinary least square, *SE* standard error, *SG* standard gamble values, *TTO* time-trade-off values, *VA* visual acuity, *WSE* worse-seeing eye.

## Discussion

This review showed a general decline in HSUVs as VA deteriorates in the BSE in AMD and DR but the relationship between VA and HSUVs was weak or absent in the WSE. This finding was not consistent across studies and this could be partly explained by the heterogeneity of included studies. No relevant studies were identified for a specified population of DMO patients. Methodological diversity was partly overcome by using a common classification used by Brown et al. [16]. However, this did not work in all cases and additionally has not been validated as the most clinically relevant classification. Although, regression models of HSUVs to VA were identified, models were not specified in the same way and little is known about the performance of the models. However, publication of regression models is most helpful as it allows the extrapolation of results to situations where the VA classification needs to be different.

Regarding anchors for utility elicitation, a majority of TTO and SG studies used perfect vision as the upper anchor rather than perfect health which suggests in a narrower scale resulting in lower scores and ranges for improvement. For economic evaluation, perfect or full generic health is recommended to enable cross programme comparison [36]. In this review, self-reported TTO and SG estimates showed a monotonic relationship to VA especially in the BSE (with one or two exceptions); the generic EQ-5D and SF-6D preference-based measures tended to lack responsiveness to changes in VA, specifically in AMD patients. Whereas SG estimates tended to be higher than TTO values in the same patients and these in turn were higher than the values from the generic instruments, the anchor points were not always the same (perfect vision versus full health). Therefore, it was difficult to compare these estimates. Furthermore, the non-responsiveness of the EQ-5D in visual conditions has been observed in previous reviews and this phenomenon has been explained by the lack of a vision-specific dimension [13,14]. One important development to overcome this limitation, is to identify condition-specific dimensions (bolt-on items or dimension extensions) to improve the usefulness of the EQ-5D in vision-related conditions [37,38]. An alternative approach would be to obtain data from patients using a preference-based version of a disease-specific instrument and one has recently become available based on the National Eye Institute Vision Function Questionnaire (NEI-VFQ 25) [39] Field test results indicate that the NEI-VFQ 25 is a reliable and valid instrument in comparing vision-related HRQoL across groups of patients [40,41]. Furthermore, a number of clinical and epidemiological studies have supported its validity in patients with AMD [40,42] and other chronic eye disorders [43-45].

An interesting finding from the literature review was the limited evidence on the relationship between HSUVs and VA in the WSE. This has important implications for the cost effectiveness modelling since clinical practice is concerned with treating the person and not just an individual eye. It would seem that having problems in the weaker eye has little or no impact on HSUVs after controlling for VA in the BSE in patients with AMD or DR. However there are significant limitations in the literature regarding the relationship between VA in the WSE or overall VA and HSUVs and this require significant work for the future.

This review is important because it provides estimates of HSUVs for defined VA levels. It also reports regression coefficients which in turn provide information on the relationship between VA and HSUVs. Although VA levels were re-classified, this was not possible for all studies. Small samples sizes of included studies introduced a significant limitation which could not be reduced by a formal meta-analysis. Statistical pooling of results was also inappropriate due to heterogeneity of relevant studies. More HSUVs measures need to be used in clinical studies in order to provide better evidence for economic evaluation.

## Conclusions

The current review presents a comprehensive overview of HSUVs in AMD and DR by VA levels reflecting a range of visual abilities. No relevant studies were identified for patients with DMO. Vision loss tends to have an obvious impact on patients’ quality of life, especially in the BSE, as shown in most of the literature. However, the relationship between VA and HSUVs is not the same between conditions or affected eyes, and is strongly related to the instrument and utility elicitation methodology used for HSUV elicitation. Most studies are concerned with VA in the BSE, but this is not always relevant for policy makers. There is very limited evidence in the WSE by VA categories.

In terms of instruments, the widely used EQ-5D does not reflect the problems associated with chronic eye conditions like AMD and DR, whereas HUI3, TTO and SG showed comparatively stronger associations with VA in the BSE. The use of a vision-specific instrument such as one based on the NEI-VFQ 25 may be a more appropriate measure of self-reported HRQoL in patients with visual disability. The QALY gains estimated in this way may better reflect the impact of the clinical intervention and the benefit observed by patients.

## Abbreviations

AMD: Age-related macular degeneration; BGV: Bilateral good vision; BSE: Better-seeing eye; CINAHL: Cumulative Index of Nursing and Allied Health Literature; DMO: Diabetic macular oedema; DR: Diabetic retinopathy; EMBASE: Electronic biomedical bibliography; Econlit: Electronic bibliography of American Economic Association; EQ-5D: EuroQol; HRQoL: Health-related quality of life; HSUV: Health state utility values; HUI3: Health utilities index mark 3; LogMAR: Logarithm of the minimum angle of resolution; MEDLINE: Medical literature analysis and retrieval system online; NEI-VFQ 25: National Eye Institute Vision Function Questionnaire - 25-item; NICE: National Institute for Health and Clinical Excellence; NR: Not reported; OLS: Ordinary least square; QALY: Quality adjusted life year; SE: Standard error; SF-6D: Short form 6D; SG: Standard gamble; TRIP: Turning research into rractice; TTO: Time trade-off; UGV: Unilateral good vision; VA: Visual acuity; VAS: Visual analogue scaling; WSE: Worse-seeing eye; WVA: Weighted visual acuity (represents 0.75 VA in BSE + 0.25 VA in WSE).

## Competing interests

EP, JC and JB work for the School of Health and Related Research that received funding from Novartis to undertake this project. AF works for Novartis and is involves in Health Economics and Outcomes Research.

## Authors’ contributions

EP contributed to the study design, managed data collection, conducted all the analyses, and developed the first draft of the manuscript and its subsequent revisions. JC assisted in data collection, analyses and interpretation of data and reviewing of drafts of the manuscript. JB designed the study, provided advice on interpretation of data and reviewed draft manuscripts. AF was responsible for conception of the study, contributed to the study design and reviewed drafts of the manuscript. All authors read and approved the final version of the manuscript.

## Pre-publication history

The pre-publication history for this paper can be accessed here:

http://www.biomedcentral.com/1471-2415/13/74/prepub

## Supplementary Material

Additional file 1 **Search strategy used in MEDLINE (Ovid).** This shows a search strategy of a combination of free text words and MESH terms used to search the MEDLINE database (Ovid MEDLINE (R) In-Process & Other Non-Indexed Citations and Ovid MEDLINE (R) 1946 to January Week 4 2012). Click here for file

Additional file 2 **Quality assessment of included studies.** This is a descriptive summary of quality assessment of included studies in the review. Quality assessment of included studies focused on the recruitment and selection of the patients together with completion rates in order to assess the representativeness of the sample of the patient group. Additional items considered were response rate to utility elicitation methods of utility elicitation as well as identifying potential confounding variables. Click here for file

Additional file 3 **Table showing utilities reported by Czoski-Murray et al., 2009 **[21]** and Espallargues et al., 2005 **[22]**.** This table displays vision-related estimates from two studies relating to age-related macular degeneration (AMD). Reported visual levels used in these studies could not be adapted using the pre-specified levels used in the study by Brown et al. [16]. Furthermore, contact lenses were used to simulated AMD visual health states in a healthy population in the study by Czoski-Murray et al., 2009 [21] while the study by Espallargues et al., 2005 [22] obtained estimates from patients with the condition. Click here for file
